# Liver Transplantation without Perioperative Transfusions Single-Center Experience Showing Better Early Outcome and Shorter Hospital Stay

**DOI:** 10.1155/2013/649209

**Published:** 2013-12-12

**Authors:** Nicolás Goldaracena, Patricio Méndez, Emilio Quiñonez, Gustavo Devetach, Lucio Koo, Carlos Jeanes, Margarita Anders, Federico Orozco, Pablo D. Comignani, Ricardo C. Mastai, Lucas McCormack

**Affiliations:** ^1^Liver Surgery and Transplantation Unit, Hospital Alemán of Buenos Aires, Avenue Pueyrredón 1640, 1118AAAT Buenos Aires, Argentina; ^2^Anesthesiology Service and Transplantation Unit, Hospital Alemán of Buenos Aires, Avenue Pueyrredón 1640, 1118AAAT Buenos Aires, Argentina; ^3^Hepatology Service and Transplantation Unit, Hospital Alemán of Buenos Aires, Avenue Pueyrredón 1640, 1118AAAT Buenos Aires, Argentina; ^4^Critical Care Unit, Hospital Alemán of Buenos Aires, Avenue Pueyrredón 1640, 1118AAAT Buenos Aires, Argentina

## Abstract

*Background*. Significant amounts of red blood cells (RBCs) transfusions are associated with poor outcome after liver transplantation (LT). We report our series of LT without perioperative RBC (P-RBC) transfusions to evaluate its influence on early and long-term outcomes following LT. *Methods*. A consecutive series of LT between 2006 and 2011 was analyzed. P-RBC transfusion was defined as one or more RBC units administrated during or ≤48 hours after LT. We divided the cohort in “No-Transfusion” and “Yes-Transfusion.” Preoperative status, graft quality, and intra- and postoperative variables were compared to assess P-RBC transfusion risk factors and postoperative outcome. *Results*. LT was performed in 127 patients (“No-Transfusion” = 39 versus “Yes-Transfusion” = 88). While median MELD was significantly higher in Yes-Transfusion (11 versus 21; *P* = 0.0001) group, platelet count, prothrombin time, and hemoglobin were significantly lower. On multivariate analysis, the unique independent risk factor associated with P-RBC transfusions was preoperative hemoglobin (*P* < 0.001). Incidence of postoperative bacterial infections (10 versus 27%; *P* = 0.03), median ICU (2 versus 3 days; *P* = 0.03), and hospital stay (7.5 versus 9 days; *P* = 0.01) were negatively influenced by P-RBC transfusions. However, 30-day mortality (10 versus 15%) and one- (86 versus 70%) and 3-year (77 versus 66%) survival were equivalent in both groups. *Conclusions*. Recipient MELD score was not a predictive factor for P-RBC transfusion. Patients requiring P-RBC transfusions had worse postoperative outcome. Therefore, maximum efforts must be focused on improving hemoglobin levels during waiting list time to prevent using P-RBC in LT recipients.

## 1. Introduction

Liver transplantation (LT) may result in significant blood loss and subsequent transfusion of red blood cells (RBCs) in most patients [1]. Although there is strong evidence supporting hemostatic defects in cirrhotic patients [2], many preoperative factors such as fulminant liver failure, bacterial infections, renal insufficiency, and severe portal hypertension may also cause imbalance in the hemostatic system. In addition, anatomical local surgical difficulties, prolonged surgical time, perioperative hypothermia, metabolic derangements, and intraoperative dilutional coagulopathy (blood transfusions and fluid administration) are factors that could potentially increase blood loss during surgery.

In the last decade, the experience acquired in the liver transplantation and management of Jehovah's witnesses patients where transfusions are not possible [3], the refinement of surgical techniques, and the appropriate anesthetic management has reduced intraoperative bleeding and the need for blood transfusions in the perioperative period during and following LT. It is widely known that there is clear association between intraoperative RBC transfusion and survival in LT [4, 5]. Certainly, significant surgical blood loss has been linked to major surgical morbidity and operative mortality, whereas RBC transfusion is associated with multiple disadvantages, risks, and increased financial burden. Furthermore, intraoperative operative blood loss independently predicts tumor recurrence and survival after radical surgery for hepatocellular carcinoma (HCC) [6].

Although the triggering variable to administer RBC is mainly hemoglobin level, today there are no uniform criteria regarding how to prevent perioperative RBC transfusion in LT recipients [7]. There is still high variability between different centers in the use of fresh frozen plasma (FFP), platelets, cryoprecipitate, fibrinogen, antifibrinolytic drugs, or desmopressin during perioperative period to prevent surgical bleeding. Other measures such as intraoperative cell saver and phlebotomy, as single or combined strategies, have been established only by few LT centers [8, 9]. As a consequence of the deleterious effect of RBC transfusion during LT, our transplant team aimed to minimize intra- and post-LT transfusion rate. Herein, we report our experience with a series of patients receiving deceased donor LT without the need for perioperative red blood cells (P-RBC) transfusion and we evaluated their outcome.

## 2. Methods

Between September 2006 and November 2011, all patients who received deceased donor LT at our unit were analyzed using a prospectively collected database. We divided the cohort in two groups according to the use of P-RBC transfusions: “No-Transfusion” and “Yes-Transfusion” (i.e., when at least one P-RBC transfusion unit was transfused). P-RBC transfusion was defined when one or more RBC units were transfused to the recipient during LT or within the first 48 hours following surgery.

The aim of our study was to assess the influence on early and long-term outcomes of using P-RBC transfusions in LT recipients. We also evaluated donor and recipient factors that could independently predict the need for P-RBC transfusions. We compared both groups according to patient, donor/graft, and perioperative variables.

### 2.1. Donor/Graft Data

Organ procurement was performed as described elsewhere with aortic and portal perfusion using University of Wisconsin preservation solution (Viaspan; DuPont, Wilmington, DE, USA) [10]. Data corresponding to donor quality were identified. Marginal grafts were defined when three or more of the following criteria coexisted: cardiac arrest >15 minutes or prolonged hypotensive episodes of <60 mm Hg for >1 hour, donor age >55 years, high vasopressor drug requirement (dopamine dose >10 *μ*g/kg/min or any doses of other amines), hypernatremia >155 mEq/L, prolonged intensive care unit (ICU) stay (i.e., >5 days with mechanic ventilation), elevated liver transaminases (AST >170 U/L or ALT >140 U/L), cold ischemia time >12 hours, warm ischemia time >40 minutes, and liver steatosis >30% [11]. Liver biopsy was performed systematically in each liver donor.

### 2.2. Recipient and Operative Data

All transplants were performed without venovenous bypass or portocaval shunt, as previously described [10]. The same surgical and anesthesiological team performed all LT. The following recipient data was collected: age, gender, history of previous upper abdominal surgery, underlying liver disease, biochemical profile, model for end-stage liver disease (MELD) score, recipient status on the waiting list (elective, emergency), surgical technique, and operative times. All intra- and postoperative transfusion requirements (RBC, FFP, cryoprecipitates, and platelets) were recorded.

Our anesthesiological strategy was focused on fluid restriction with low central venous pressure (CVP) during surgery. Maintenance fluids and crystalloids were administered to stabilize blood pressure >90 mmHg and ensure diuresis of at least 0.5 mL/kg/h. When fluid restriction was ineffective to keep a low CVP, vasoactive agents were used.

### 2.3. Postoperative Outcome

Liver allograft function was evaluated clinically and through biochemical parameters such as aspartate aminotransferase (AST), alanine aminotransferase (ALT), and bilirubin and prothrombin time measured daily during the first week. Liver graft vascular patency was evaluated by echo-Doppler ultrasound during the first day and when clinically indicated. Primary nonfunction (PNF) of the graft was defined as death or retransplantation within 7 days following LT in the absence of any vascular problems. Primary dysfunction (PDF) of the graft was assumed when a peak AST level >1.500 IU/L and a prothrombin time <50% cooccurred within the first week of LT [12]. Postoperative major complications included complications of grades 3–5 (i.e. requiring surgical intervention or ICU admission or causing death, resp.) according to a validated classification system for postoperative complications [13]. Postoperative bacterial infection was defined as any clinical sign of infection in conjunction with positive bacteriological cultures within 30 days after surgery. Postoperative overall infection was defined as any documented infection (i.e., viral, bacterial, or fungal) with positive serology or cultures within 30 days after surgery.

After discharge, each patient was followed in the multidisciplinary outpatient clinic. Hepatocellular carcinoma and hepatitis C virus (HCV) recurrence were studied in each patient as recommended [14, 15]. All HCV recipients underwent 1-year protocol liver biopsy as part of our routine practice for early HCV recurrence diagnosis. HCV recurrence was considered when liver fibrosis ≥1 METAVIR score was present [16]. Long-term outcome was analyzed using 1- and 3-year patient survival rates.

### 2.4. Statistical Analysis

Summary data are presented as median (range). Differences between groups were tested by chi-squared test for categorical and Mann-Whitney *U*-test for continuous variables. All tests were two-tailed. *P* < 0.05 indicated statistical significance. Patients' survival was analyzed by the Kaplan-Meier method. The outcome event for patient survival was “death” or “alive.” Comparisons between survival curves were performed using the log-rank test. Calculations were performed using SPSS Version 13.0 (SPSS, Inc., Chicago, IL, USA). Multivariate analysis was performed using a logistic regression model to assess which factors were independently related to the need for P-RBC transfusions.

## 3. Results

During the 62-month study period, 235 patients were included in our LT waiting list and, finally, 127 were successfully transplanted. Among them, 46 patients (36%) did not receive any intraoperative RBC transfusion but 7 of them were finally transfused with RBC units after the operation. Therefore, 39 (31%) patients did not receive any P-RBC transfusions constituting the “No-Transfusion” group, and 88 (69%) patients constituted the “Yes-Transfusion” group.

### 3.1. Recipient's Preoperative Status

Both groups were comparable regarding age, gender, body mass index (BMI), history of previous upper abdominal surgery, cause of cirrhosis, diagnosis of HCC, diagnosis of HCV infection, and waiting list status (Table 1). Although median MELD score was significantly higher in “Yes-Transfusion” group (11 versus 21; *P* < 0.0001), the number of patients with MELD score ≥25 was equivalent in both groups. Moreover, only few patients received MELD priority points due to early HCC in both groups (4 patients in the “No-Transfusion” and 5 in the “Yes-Transfusion”). Preoperative biochemical profile showed that hematocrit, hemoglobin level, platelet count, and prothrombin time levels were significantly lower in “Yes-Transfusion” group when compared to the “No-Transfusion” group (Table 2).

### 3.2. Graft and Donor Quality

The number of patients transplanted using marginal grafts was equivalent in “No-Transfusion” and “Yes-Transfusion” groups (21 versus 10%, resp.; *P* = 0.15). In addition, variables such as cold and warm ischemia times and the presence of liver steatosis in each liver donor were equally distributed in both groups (Table 3).

### 3.3. Operative Variables and Transfusion Requirements

A full-size liver was implanted in 113 patients (89%) and only 10 patients received a split liver graft. The technique for LT was equal in both groups (Table 4). As expected, median operative time (227 versus 240 min; *P* = 0.02) and blood components transfusion were higher in the “Yes-Transfusion” group (Table 4). To note, intraoperative transfusion of RBC was not needed in 36% of our LT patients. Aprotinin infusion, cell saver, or phlebotomies were not used to reduce intraoperative bleeding or RBC transfusions in any circumstances.

### 3.4. Early Postoperative Outcome

Only one patient developed PNF and died after LT (Table 5). The incidence of PNF, PDF, major complications, and biliary complications was the same in both groups (Table 5). However, hemodialysis need (0 versus 10%; *P* = 0.01), bacterial infections (10 versus 27%; *P* = 0.03), and postoperative overall infection rate (5 versus 22%; *P* = 0.02) were significantly higher in the “Yes-Transfusion” group (Table 5). Median ICU (2 versus 3 days; *P* = 0.003), hospital stay (7.5 versus 9 days; *P* = 0.01), and prolonged hospital stay >15 days (10 versus 27%; *P* = 0.03) were also significantly higher in the group of patients needing P-RBC. Although 30-day mortality rate was higher in the “Yes-Transfusion” group (10 versus 15%), this difference was not significant (Table 5).

### 3.5. Long-Term Outcome

**Figure 1:**
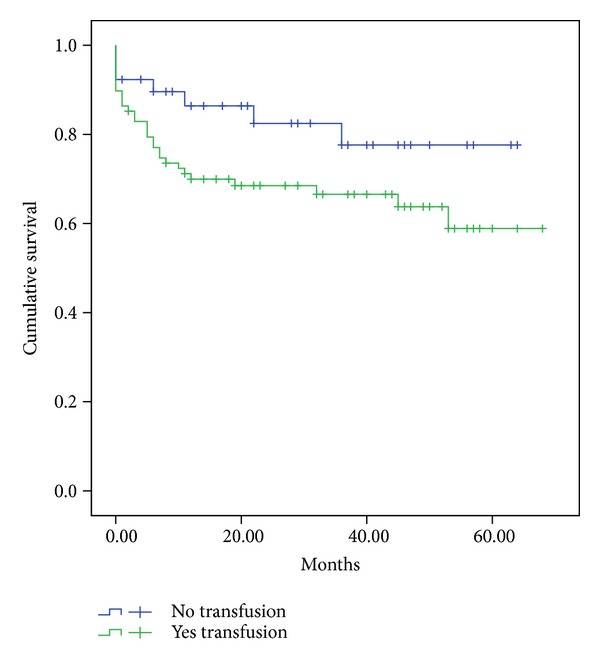
Survival curve after LT for the No- and Yes-Transfusion groups. Legend**: **1- (86 versus 70%) and 3-year (77 versus 66%) patient survival is similar in the No- and Yes-Transfusion groups, respectively (*P* = 0.09).

HCV recurrence was equal in both groups. Interestingly, HCC recurrence after LT was only observed in the “Yes-Transfusion” group (0 versus 6 patients; *P* = 0.12), but without statistical relevance. Although one- (86 versus 70%; *P* = 0.09) and 3-year survival rates (77 versus 66%; *P* = 0.09) were better in the “No-Transfusion” group, this difference was not statistically significant (Figure 1).

### 3.6. Multivariate Analysis

All preoperative donor, graft, and recipient data were included in a univariate analysis to determine variables that were unequally distributed in both groups of patients. Each significant variable was analyzed using a logistic regression model to assess which factors were independently associated with the need for P-RBC transfusions. Baseline patient's hemoglobin level before surgery (*P* < 0.001) was the unique independent preoperative risk factor associated with P-RBC requirement. Surprisingly, extended donor criteria, graft steatosis, and MELD score were not a predictive factor for P-RBC transfusion in our series.

## 4. Discussion

The need for blood transfusion therapy has remained a critical feature in LT. In contrast with transplantation of other organs, the intrinsic coagulopathy defects of LT candidates and the frequent presence of severe portal hypertension make transfusion-free surgery a major challenge [17]. Moreover, there is minimal consensus on transfusion guidelines during or after LT [7]. Most studies have focused on the deleterious effect of intraoperative massive blood transfusion without putting emphasis on the importance of avoiding transfusions in the early phase after LT [4, 9, 18, 19]. To the best of our knowledge, this is the first study investigating the influence of using P-RBC on early and long-term outcomes after LT. We observed that early outcome after LT is better with reduced bacterial infections, hemodialysis need, and overall infection rate when P-RBC transfusions are avoided. Surprisingly, neither donor/graft quality nor recipients MELD score were predictive factors associated with the need for P-RBC transfusions. As hemoglobin level was the only predictive factor in our cohort, maximum efforts should be placed on improving anemia in patients waiting for LT.

Many strategies have been proposed for reducing intraoperative bleeding including the maintenance of a CVP ≤ 5 cm of water, reverse Trendelenburg position, systemic infusion of nitroglycerin, and the use of antifibrinolytics agents, recombinant factor VIIa, or aprotinin [7–9]. Unfortunately, the clinical use of aprotinin has been questioned due to an increased incidence of renal failure, stroke, and myocardial infarction and it has been withdrawn from the market [20]. One Canadian group proposed using phlebotomy as an interesting strategy to reduce RBC requirements [8]. Three randomized controlled trials (RCT) demonstrated that the routine use of recombinant factor VIIa for patients undergoing LT is not recommended [21]. Our policy based on maintenance of low CVP was used safely with minor hemodynamic disturbance in most patients. However, to achieve this goal, a close communication between the surgical and the anesthesia team is crucial during transplant to delineate perioperative interventions targeted to minimize blood loss.

Several studies have looked into factors that can predict transfusion requirements [9, 22–24]. One study demonstrated a relationship between the starting platelet count, duration of surgery, the starting INR value, and the number of RBC units that were transfused during LT [22]. Current organ allocation system in Argentina is based on giving priority to the patients with the highest MELD scores [25, 26]. Usually, patients with high MELD scores have poor coagulation status with reduced INR values and low platelet count related to hypersplenism. Although it could be expected that the need for intraoperative RBC transfusion can be predicted by the preoperative prothrombin time and degree of thrombocytopenia, we failed to confirm this in our cohort. We observed that low baseline hemoglobin level was the only independent predictor for P-RBCs transfusion during LT. In contrast with a previous study, we observed that donor and graft quality did not influence the need for RBC during LT [27]. Hence, we believe that extramedical effort must be made to improve hemoglobin levels during waiting list time. In this scenario, many strategies can be considered during the waiting time so as to improve hemoglobin levels, such as administration of eritropoyetin, iron, and folinic acid, and in some situations even considering the use of RBC transfusions during waiting time to patients with hemoglobin levels ≤ 7 mg/dL [28]. However, none of these strategies have been correctly validated in the field of LT and further analysis with well-designed RCT is needed to support them.

Unfortunately, the current literature review is unclear about the exact incidence of blood transfusions in LT. While some reported routine RBC transfusions during LT [1], others made maximum efforts to minimize blood loss [29]. Furthermore, there may be a bias towards underreporting due to lack of clear definitions of the “perioperative period” in this context and, perhaps, disinterest in the medical community on this topic. However, the relationship between immunocompetence during the perioperative period and recurrence-free survival after LT is becoming a topic of interest, especially for patients with HCV infection or HCC. Probably because this study is based on a small series, we failed to demonstrate any negative effect on viral or tumor recurrence in patients needing P-RBC. The effect of novel anesthetic techniques and perioperative management on positively influencing the balance between inflammation and immune competence is an intriguing avenue for future studies. Thus, we urge transplant community to start reporting data on blood transfusions and to study its impact on clinical outcomes in patients undergoing transplant surgery.

Apart from the obvious intraoperative life-saving benefits, there is accumulating evidence that RBC transfusions are associated with substantial complications after LT [9, 18]. The risk of allogeneic blood transfusion extends beyond viral transmission and includes allergic reactions, alloimmunization, bacterial sepsis, transfusion-related acute lung injury, renal failure, excessive intravascular volume, and immunosuppressive effects. However, data are only related to the administration of blood components during surgery and scarce data has been published concerning its use during early postoperative time after LT. As probably intra- or early postoperative RBC transfusion could have a similar impact on outcome and considering that probably the reasons for differences on the administration timing or location could be mainly logistic, we decided to analyze transfusions during and within 48 hours after surgery. Interestingly, we found that only few patients were transfused after surgery demonstrating some kind of agreement between anesthesiologist and ICU doctors. In agreement with others, we observed that postoperative complication in terms of infections and hemodialysis need was increased in transfused patients [30]. We additionally confirmed that ICU and hospital stay are longer in patients needing P-RBC transfusions. In the future, a cost analysis of our RBCs-saving strategy will probably provide economic arguments for reducing perioperative transfusions that should be weighed against patient safety.

In conclusion, poor donor/graft quality and high recipient MELD score are not a predictive factor for P-RBC transfusion during LT. The only independent predictive risk factor is the baseline preoperative hemoglobin level. Patient requiring P-RBC transfusions had more complications in terms of higher infections and hemodialysis need, prolonging ICU and hospital stays. Maximum efforts must be focused on developing novel strategies for improving hemoglobin levels during waiting list time to improve early outcome after transplantation.

## Figures and Tables

**Table 1:** Recipient preoperative status in No- and Yes-Transfusion groups.

	No Transfusion *n* = 39	Yes Transfusion *n* = 88	*P*
Age (years)*	57 (18–73)	56 (1–72)	0.99
Male (%)	26 (67)	54 (61)	0.69
BMI	22 (19–35)	22 (15–35)	0.99
Previous upper abdominal surgery (%)	8 (20)	13 (15)	0.42
Elective/emergency	35/4	76/12	0.77
MELD*	11 (6–26)	21 (6–50)	0.0001
MELD ≥ 25 (%)	5 (13)	25 (28)	0.07
HCC (%)	8 (21)	25 (28)	0.20
HCV cirrhosis (%)	14 (36)	18 (20)	0.07

*Median and Range; BMI: body mass index; MELD: model for end-stage liver disease; HCC: hepatocarcinoma; HCV: hepatitis C virus.

**Table 2:** Preoperative biochemical profile of patients receiving LT.

	No Transfusion *n* = 39	Yes Transfusion *n* = 88	*P*
Hemoglobin (g/dL)*	12.5 (8.3–19.3)	10 (6.1–14.3)	0.0001
Hemoglobin < 8 g/dL (%)	0	13 (15)	0.009
Hematocrit (%)	35 (24–54)	29.4 (19–41)	0.0001
Hematocrit < 25% (%)	1 (3)	20 (23)	0.004
Platelet count (100 × 10^9^/L)*	98 (15–461)	81.5 (10–470)	0.051
Platelets < 100 × 10^9^/L (%)	18 (46)	59 (67)	0.03
Prothrombin time (%)*	62 (25–100)	44 (6–100)	0.0001
Prothrombin time < 50% (%)	14 (36)	52 (59)	0.02

*Median and range; LT: liver transplantation.

**Table 3:** Graft and donor quality variables in the two groups of LT patients.

	No Transfusion *n* = 39	Yes Transfusion *n* = 88	*P*
Marginal graft (%)	8 (21)	9 (10)	0.15
Cold ischemia time (min)*	366 (206–1180)	380 (188–830)	0.11
Warm ischemia time (min)*	35 (21–45)	37.5 (27–65)	0.53
Graft steatosis > 30% (%)	5 (12.8)	8 (9)	0.53

*Median and range; LT: liver transplantation.

**Table 4:** Intraoperative variables and blood products transfusion in the cohort of LT patients.

	No Transfusion *n* = 39	Yes Transfusion *n* = 88	*P*
Piggyback LT technique (%)	2 (5)	7 (8)	0.9
Full-size/split LT	39/0	78/10	0.06
Operative time (min)*	227 (135–320)	240 (150–420)	0.02
Intraoperative RBC*	0	2 (0–6)	0.0001
Intraoperative plasma*	6.6 (0–14)	9 (0–16)	0.0001
Intraoperative cryoprecipitates*	0 (0–7)	0 (0–9)	0.28
Intraoperative platelets*	0 (0–8)	0 (0–15)	0.024
Perioperative RBCs transfusion*	0	3.5 (1–16)	0.0001

*Median and range; LT: liver transplantation; RBCs: red blood cells.

**Table 5:** Postoperative outcome following LT in No- and Yes-Transfusion groups.

	No Transfusion *n* = 39	Yes Transfusion *n* = 88	*P*
PNF (%)	0	1 (1)	1
PDF (%)	8 (21)	18 (20)	1
30 days—arterial thrombosis	0	0	1
AST peak (U/L)*	784 (154–17600)	859 (132–11830)	0.76
ALT peak (U/L)*	639 (162–4680)	688 (139–4890)	0.061
ICU stay (days)*	2 (1–11)	3 (0–65)	0.003
Hospital stay (days)*	7.5 (5–21)	9 (2–105)	0.01
Hospital stay ≥ 15 days (%)	4 (10)	24 (27)	0.03
30-day mortality (%)	4 (10)	13 (15)	0.58
Major complication (%)	7 (18)	30 (34)	0.9
Hemodialysis need (%)	0	9 (10)	0.01
Bacterial infections (%)	4 (10)	24 (27)	0.03
Infections within 30 days (%)	2 (5)	19 (22)	0.02
Biliary complications (%)	3 (8)	3 (3)	0.37
VHC recurrence (≥F1) (%)	8 (57)	9 (50)	0.68
HCC recurrence (%)	0	6 (24)	0.12
1-year survival %	86	70	0.09
3-year survival %	77	66	

*Median and range; PNF: primary nonfunction; PDF: primary dysfunction; AST: aspartate aminotransferase; ALT: alanine aminotransferase; ICU: intensive care unit; HCV: hepatitis C virus; HCC: hepatocarcinoma.

## Abbreviations

LT: Liver transplant
RBCs: Red blood cells
HCC: Hepatocellular carcinoma
FFP: Fresh frozen plasma
P-RBC: Perioperative red blood cells
ICU: Intensive care unit
MELD: Model for end-stage liver disease
CVP: Central venous pressure
AST: Aspartate aminotransferase
ALT: Alanine aminotransferase
PNF: Primary nonfunction
PDF: Primary dysfunction
HCV: Hepatitis C virus
BMI: Body mass index
RCT: Randomized controlled trials.
